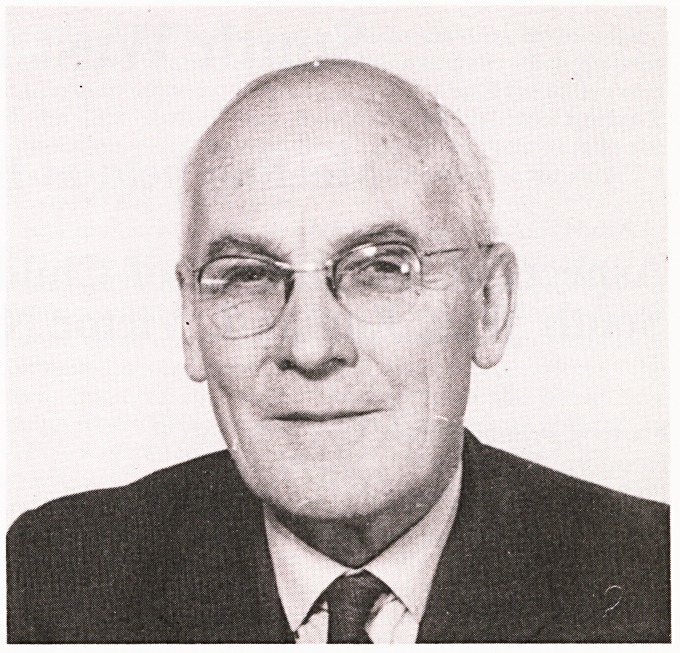# Dr Gordon Heron

**Published:** 1990-03

**Authors:** 


### Obituary

### Dr GORDON HERON

Alexander Gordon HERON M.B., Ch.B., F.R.C.G.P. who died on 1st September 1989 at the age of 88 years was for many years one of the elder statesman of medicine in Bristol.

Medicine was in his blood for his grand-father was, in his own words, a doctor-farmer in the County Down of Northern Ireland while his father was a general practitioner in Bristol for 53 years.

Gordon was born in Bristol in 1901, went to school at Clifton College and received his medical training at Bristol Royal Infirmary. He was a keen sportsman and played rugby for Bristol University for five years and was captain in his last year. Thereafter he represented West Gloucestershire at Hockey until the outbreak of war.

He qualified in medicine in 1924 and was doing his resident House appointments at BRI when in March 1925 there was a severe epidemic of influenza in Bristol and his father who was in practice in the Gloucester Road appealed for his help. He was allowed to leave B.R.I. immediately to join his father and worked continuously in the same practice for the next 52 years.

In 1926 he married Doris Joscelyne a young doctor who had been a fellow student at B.R.I. and in 1929 he took into partnership her brother Pat Joscelyne and together they built up a very successful general practice in North Bristol.

When war came in 1939 Pat, who was in the Territorial Army had to leave immediately to join the R.A.M.C., leaving Gordon to carry the whole burden of the practice himself. He was appointed an emergency anaesthetist to the Orthopaedic Department at B.R.I. and had to report for duty every time that Bristol was bombed. He also joined the Home Guard and was responsible for raising and training first-aid teams and stretcher bearers throughout the war. He had many exciting experiences during the Bristol air-raids and there is no doubt that he had as active and dangerous a war as many doctors in the Armed Forces.

By the time the war ended in 1945 he had been qualified for twenty years and was a senior and very experienced doctor and not surprisingly took an active part in local medical politics—particularly when the National Health Service started in 1948. He was a loyal member of the B.M.A. and for twenty years from 1947 and 1967 he represented Bristol General Practitioners on the B.M.A. Representative Body. He was President of the Bristol Division in 1961 and was made a Fellow of the B.M.A. in 1964. He became a Founder member of the Royal College of General Practitioners and was made a Fellow of that College in 1971. For many years he was a member of the Bristol Executive Council and of the Bristol Medical Committee of which he was Chairman in 1962. He served also on the South Western Regional Hospital Board and on the Bristol University Court.

In 1954 Gordon was President of the Bristol Medico-Chirurgical Society and his Presidential address entitled "That Backbone" was a vigorous and eloquent plea for the upgrading of general practice which was at a low ebb in the early days of the National Health Service. He advocated the introduction of medical students into general practice experience well before they actually qualified and also the appointment of general practitioners as Clinical Assistants to Consultants in Teaching Hospitals—both ideas which were soon accepted and widely and adopted throughout the country.

He was closely involved in the development of post-graduate education and continued to take an active part in local medical politics—but above all he was a dedicated and greatly admired general practitioner. As he said in his Presidential address—a general practitioner must be father, friend and confessor to his patients and an adviser on almost anything under the sun—and this Gordon was in the fullest possible sense.

He had a wide circle of friends and was for many years a member of the Bristol Medical Reading Society. He was an active Freemason and reached high rank both locally and nationally.

Eventually in 1977 at the age of 76 years he retired from active practice after 52 years of hard and dedicated and rewarding work. His marriage to Doris was supremely happy and in 1976 they celebrated their Diamond Wedding. He is survived by Doris and by his two sons and his daughter, who is herself a doctor, and by seven grand-children and five great-grand-children. A.T.M.R.

## Figures and Tables

**Figure f1:**